# Developing a model to assess community-level risk of oral diseases for planning public dental services in Australia

**DOI:** 10.1186/s12903-016-0200-5

**Published:** 2016-03-31

**Authors:** Andrea M. de Silva, Panagiota Gkolia, Lauren Carpenter, Deborah Cole

**Affiliations:** Centre for Applied Oral Health Research, Dental Health Services Victoria, 720 Swanston Street, Carlton, 3053 Australia; Melbourne Dental School, University of Melbourne, Carlton, 3053 Australia; Infectious Diseases Division, Department of Internal Medicine I, University Hospital Tübingen, Otfried-Müller-Street, Tübingen, 72076 Germany; Jack Brockhoff Child Health and Wellbeing Program, Centre for Health Equity, The Melbourne School of Population and Global Health, University of Melbourne, Bouverie Street, Carlton, 3053 Australia; Dental Health Services Victoria, 720 Swanston Street, Carlton, 3053 Australia

**Keywords:** Dental, Health services, Planning, Data

## Abstract

**Background:**

Poor oral health is a chronic condition that can be extremely costly to manage. In Australia, publicly funded dental services are provided to community members deemed to be eligible—those who are socio-economically disadvantaged or determined to be at higher risk of dental disease. Historically public dental services have nominally been allocated based on the size of the eligible population in a geographic area. This approach has been largely inadequate for reducing disparities in dental disease, primarily because the approach is treatment-focused, and oral health is influenced by a variety of interacting factors. This paper describes the developmental process of a multi-dimensional community-level risk assessment model, to profile a community’s risk of poor oral health.

**Methods:**

A search of the evidence base was conducted to identify robust frameworks for conceptualisation of risk factors and associated performance indicators. Government and other agency websites were also searched to identify publicly available data assets with items relevant to oral diseases. Data quality and analysis considerations were assessed for the use of mixed data sources.

**Results:**

Several frameworks and associated indicator sets (twelve national and eight state-wide data collections with relevant indicators) were identified. Determination of the system inputs for the Model were primarily informed by the World Health Organisation’s (WHO) operational model for an Integrated Oral Health-Chronic Disease Prevention System, and Australia’s National Oral Health Plan 2004–2013. Data quality and access informed the final selection of indicators.

**Conclusions:**

Despite limitations in the quality and regularity of data collections, there are numerous data sources available that provide the required data inputs for community-level risk assessment for oral health. Assessing risk in this way will enhance our ability to deliver appropriate public oral health care services and address the uneven distribution of oral disease across the social gradient.

## Background

In Australia, publicly funded dental services are provided free or at little cost only to community members who are socio-economically disadvantaged, or at higher risk of developing dental disease for other reasons. The historical approach of allocating public dental services nominally on the size of the eligible population in a geographic region has been largely inadequate for reducing disparities in dental disease. Further, the approach has generally focused on treatment needs rather than the causes of oral diseases, with the role of public dental services in disease prevention remaining under-developed. Oral health should also not be considered in isolation however, and to reduce the social gradient in the prevalence of dental disease, the underlying causes of ill health more broadly also need to be considered.

Whitehead identifies that health is influenced by individual lifestyle factors; social and community networks; living and working conditions; and socio-economic, cultural and environmental conditions [1]. The causes of social inequality in health are considered to be multiple and inter-related, which therefore requires that actions to address the issue must be interconnected across the different levels of influence. Further, the socio-ecological framework proposed by McLeroy et al. [2] identifies that numerous systems and contexts shape human development [3, 4]. As such, the outcomes of both whole system, and more focussed interventions will depend upon factors operating at multiple levels. Points of intervention exist at the policy, community, organisational, interpersonal and intrapersonal levels [5].

There has been some conceptualisation of the social determinants of oral health, and the factors that operate and interact at multiple levels to influence oral health [6]. However, there has been little translation of the conceptual frameworks into a tangible mechanism to drive decision-making in public dental services. The question remains as to how public oral health services, and the broader public oral health care system, should be re-oriented to reduce inequities in oral health using an evidence-based, public health approach.

The aim of this paper is to describe the developmental process for a community-level risk assessment model for oral health to inform the service-delivery approach of Dental Health Services Victoria (DHSV) into the future.

## Methods

### Stakeholder consultation

DHSV is the leading public oral health care provider in Victoria, which is the second most populous state in Australia. In 2013–14, through the Royal Dental Hospital of Melbourne and over 90 community dental clinics, DHSV provided public oral health services to 411,217 Victorians. However, to enhance the service provided, reduce health inequities, and ensure future activities are appropriately oriented, an evidence-informed, public health approach to planning is to be utilised.

Within DHSV, consultation with a broad range of stakeholders identified that although risk assessment for oral disease is routinely performed at an individual-level in the dental clinic, to develop a state-wide service and public oral health care system, it is necessary to develop a method of assessing risk of oral disease at a community-level. Such a community risk assessment model would need to acknowledge the multiple factors influencing oral health, as well as the broader social determinants of health. To operate at a service and system-level, the model would also need to be updateable with new data as it becomes available, allow ongoing monitoring and surveillance, have capacity to examine geographical variation in oral health needs, and have utility to inform the optimal allocation of public oral health care services across Victoria.

**Fig. 1:**
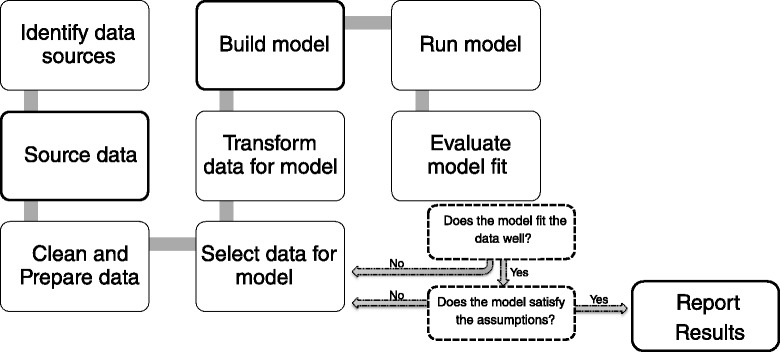
Flow chart shows steps in model development and testing

This project aims to use the best research available to develop a multi-dimensional, community-level risk model which predicts the oral health needs in a given community. The profile would then identify which aspects of the oral health care system should be improved. An overview of the entire process to be undertaken is provided in Fig. 1; this paper reports on the first two steps in the process.

### Identification of guiding theoretical frameworks

An important aspect of the developmental stage was to identify a robust and evidence-based theoretical framework to guide the selection of appropriate indicators and data sources. A comprehensive literature review of published frameworks for risk factors of oral health for children and adults and associated indicators was conducted. The Ovid Medline bibliographic database was searched for relevant articles using the following terms and their combinations: ‘frameworks’, ‘indicators’, ‘oral health survey’, ‘oral health monitoring’, ‘epidemiological data’, ‘modelling’, ‘dental need’, and ‘population data’. Health Department and other government websites were also searched for documents relating to oral or dental health monitoring.

### Identification of available data sources

The following sources were examined to identify existing Australian data collections for use in the statistical modelling: websites of offices of national statistics; national and state-wide population health surveys (interview and examination); longitudinal cohort studies, and surveillance networks.

There was a particular focus on identifying data sources with participants comparable to (or comprising) the Victorian population and where data was collected as part of health surveys, or as part of clinical or administrative data collections. Oral health examination and interview surveys restricted solely to population groups with special needs were excluded from further consideration.

### Selection of indicators and assessment of data quality

The selection of the indicator set was guided by consistency with underpinning theoretical frameworks, current oral health policy priorities, and scientific validity, reliability and relevance. The Australian Bureau of Statistics (ABS) report ‘Measuring Wellbeing: Frameworks for Australian Social Statistics 2001’ [7] underlines these basic measurement and analysis issues associated with available data sources in Australia. Inclusion of an indicator in the risk model was also determined after assessing the basic data quality dimensions of: methodological soundness, accessibility, accuracy and reliability [8, 9], as described below.

#### Methodological soundness

The methodological basis for the statistics follows internationally accepted standards, guidelines, or good practices.

- Concepts and definitions used are in accord with internationally-accepted statistical frameworks.
- The scope is in accordance with internationally accepted standards, guidelines, or good practices

#### Accessibility

Data and metadata are easily available and assistance to users is adequate

- Statistics are presented in a clear and understandable manner, forms of dissemination are adequate, and statistics are made available on an impartial basis.
- Up-to-date and pertinent metadata are made available.

#### Accuracy and reliability

Source data and statistical techniques are sound and statistical outputs sufficiently portray reality.

- Source data available provide an adequate basis to compile statistics
- Statistical techniques employed conform to sound statistical procedures

### Ethics and consent

As no data was collected for this study no ethics approval or participant consent was sought.

## Results

### Identification of frameworks

**Fig. 2:**
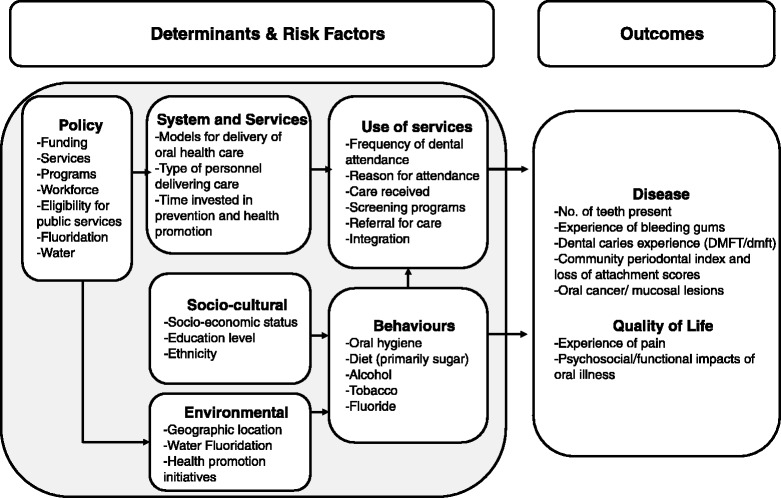
Multi-level framework of oral health risk factors and determinants

Several multi-level frameworks were identified through the searching. The most relevant for this project were: Australia’s National Oral Health Plan 2004–2013 [10], The Socio-Ecological Model of Health [2], Fisher-Owens Model of Child Oral Health [11], WHO model for Oral Health Diseases Surveillance and WHO operational model for an integrated oral health-chronic disease surveillance system [9] and the Framework of Socioeconomic Determinants of Health [12]. The system inputs for our community-level risk assessment model were developed primarily around the WHO frameworks [9, 13, 14] and Australia’s National Oral Health Plan 2004–2013 [10]. The framework encompasses two ‘Tiers’, covering Determinants and Risk factors, and Outcomes. Each tier has a number of domains, each defining a distinct aspect of the tier. The Determinants and Risk factors tier has six domains that bring together a range of factors that affect oral health at individual and population levels: Policy, Health Systems, Sociocultural, Environmental, Behaviours, and Use of Services. The Outcomes tier has two domains that summarise the impact of oral health conditions on individuals: Disease and Quality of Life. The framework is depicted in Fig. 2.

The Australia’s National Oral Health Plan 2004–2013 [10] encompasses nineteen process and outcomes indicators for Oral Health under the key domains of the WHO framework [10], which address the main dental diseases: dental caries, periodontal diseases, and oral cancer.

### Identification of data sources

Identified national and state-wide sources with items relevant to oral health status and outcomes indicators, diseases and related risk factors were of two main categories: statistical sources (including surveys and censuses); and administrative sources (including population registries and hospital databases).

A wide range of administrative systems and statistical data collections were compiled by the Australian Institute of Health and Welfare (AIHW) from data supplied by states and territories. Other national sources were: the Australian Bureau of Statistics (ABS), the Department of Human Services (DHS), the Department of Families, Housing, Community Services and Indigenous Affairs (FaHCSIA), and the Australian Institute of Family Studies (AIFS).

Majority of identified state-wide data collections were sourced from the Department of Health and Human Services (DHHS) and Dental Health Services Victoria (DHSV) Other sources were the Cancer Council Victoria (CCV) and the Victorian Department of Education and Training (DET).

The complete list of data sources is summarised in Table 1.

**Table 1:** Audit of available data sources and assessment of data quality

Data Source	Data Set	Methodological Soundness	Accessibility	Accuracy and Reliability
Australian Bureau of Statistics (ABS)	Census [19] Population-level data on socioeconomic variables and demographics for a variety of geographic regions	Snapshot of Australian general population	Data collected every 5 years and freely available Most recent census data for 2011	Self-reported data Main sources of error: respondent error (self-enumerated), partial or non-response, processing error Quality management procedures applied to reduce error
Department of Human Services (DHS)	Health Care Card data [20] National data collected by the government welfare agency ‘Centrelink’ of adults and children issued a low income ‘Health Care Card’ which provides access to health care and related benefits, including lower costs.	General Australian population	Routinely collected data available by geographic location	Administrative records data
Australian Institute of Health and Welfare (AIHW); and AIHW Dental Statistics and Research Unit (DSRU)	National Health Workforce Data Set (NHWDS) [21] Combines data from the National Registration and Accreditation Scheme (NRAS) with data collected from the Dental Workforce Survey (DWS)	All registered dental practitioners	Available data for 2011 and 2012 Access restricted to statistical reports by AIHW	Registration data together with survey data In deriving estimates two sources of non-response to survey are accounted for: item non-response and survey non-response
	Australian Cancer Database (ACD) [18] Data collection of all primary, malignant cancers diagnosed in Australia since 1982. The ACD is compiled at the AIHW from cancer data provided by state and territory cancer registries through the Australasian Association of Cancer Registries. These registries receive information on cancer diagnoses from a variety of sources such as hospitals, pathology laboratories, radiotherapy centres	Population –based cancer registered patient data in Australia	Data released in AIHW publications and available as interactive data AIHW can also make available a broad range of cancer statistics subject to scientific/ethical review process	Weighted estimates Clinical data based on medical diagnosis
	National Hospital Morbidity Database (NHMD) [22] Compiled from data supplied by the State and Territory health authorities. It is an electronic collection of records for separations (episodes of care) in public and private hospitals in Australia. It contains demographic, administrative and length of stay data and data on diagnoses of the patient and medical procedures.	Representative general population level data for all separations of admitted patients from all public and private national hospitals	Ongoing collection Statistical reports by AIHW Data requests subject to ethics approvals	Clinical data based on medical diagnosis Extensive validation of data Data are checked for valid values, logical and historical consistency
	National Survey of Adult Oral Health (NSAOH) 2004-2006 [15] A descriptive ‘snapshot’ of oral health in the adult population of Australia. Random sample of the general population residing in all Australian states and territories. Information is collected using interviews and standardised dental examinations. The survey aims to describe levels of oral disease, perceptions of oral health and patterns of dental care within a representative cross-section of adults across Australia.	General population 15+ years old Sample size: 2267 Response rate: 44 % of sampled population Three-stage stratified sampling design Oral examination restricted to dentate population (*N =* 1181; 50 % of eligible)	Incidental data Access restricted to statistical reports by AIHW/DSRU	Self-reported oral health status data and clinical data (dentate population) Computer assisted interviews, structured questionnaires pilot tested, trained interviewers Reporting bias Sampling weights applied
	National Dental Telephone Interview Survey (NDTIS) [16] Telephone survey of a random sample of the Australian population. Respondents include users and non-users of dental services and people eligible and not eligible for public-funded dental care. NDTIS collects basic features of oral health and dental care within the Australian population, including access to services. There is no clinical component to the survey. Survey aims to: collect oral health and dental care data within the Australian population; monitor the extent of social inequalities within the dental sector; investigate the underlying reasons behind dental behaviours, and the consequences of these behaviours.	General population 5+ years old. Two-stage stratified sample design. Sample size varies between surveys.	Every 2 ½ years Access Restricted to statistical reports by AIHW/DSRU	Self-reported survey data Computer assisted interviews, structured questionnaires pilot tested, trained interviewers Reporting bias Sampling weights applied
	Child Dental Health Survey (CDHS) [16] The Survey monitors the dental health of children enrolled in school and community dental services operated by the health departments or authorities of State and Territory governments. Survey aims to: examine the distribution of oral health status by geographic location and demographic factors, and the identification of high-risk groups; and examine changes in oral health status among children over time	Random sample of children 4–15 years old enrolled in the school dental services Sample size varies between surveys	Annual data collection Data available for Victoria up to 2004 Access restricted to statistical reports by AIHW/DSRU	Routine clinical examination data. Inter/intra examiner reliability not assessed Data cleaning processes to correct data entry errors and eliminate duplicate cases Sampling weights applied
	Child Oral Health Study (COHS) 2002-2004 [17] A Survey of parents of children from South Australia, Victoria, Tasmania and Queensland	Random sample of children aged between 5 and 15	Incidental data Access restricted to statistical reports by AIHW/DSRU	Retrospective reporting Recall bias Data weighted to the age, sex and estimated resident populations
	Longitudinal Survey of Dentists’ Practice Activity (LSDPA) [23] A survey of Australian dentists that report on services provided in private dental practice. LSDPA data were used to estimate the mean services and cost of services for each visit reported in NDTIS.	Random sample of dentists from the dental register surveyed at five-year intervals. Response rate 70 % + at all five waves. Sample supplementation procedure provided representative cross-sectional sample	Five yearly-survey commenced in 1983–84 and completed in 2009–10 Statistical reports by DSRU/AIHW	Mailed structured self-completed questionnaires Validation study indicated representative estimates Weighted estimates
	National Dental Labour Force Data Collection (NLFDC) [24] The National Dental Labour Force Survey collects information on the demographic and employment characteristics of dental practitioners in Australia. Data comes from the National Registration and Accreditation Scheme (NRAS) and optional Dental Workforce Survey	All dental practitioners registered in Australia at the time of the survey	Response rates vary between years and States Significant delays between data collection and release of statistics Most recent reports in 2006 and 2009	Item on-response and survey non-response Data are weighted to account for the population being examined and imputed for non-response questions
Cancer Council Victoria (CCV)	Victorian Cancer Registry (VCR) [34] Population-based cancer registry receiving information on cancer diagnoses from 240 hospitals, 30 pathology laboratories and cancer screening services in Victoria	All cancer diagnosis in Victorian residents	Online summary statistics (incidence and mortality) by type of cancer, age, sex, year of diagnosis, region of residence Data can also be requested (conditions and limitations apply)	Clinical data based on medical diagnosis
Cancer Council Victoria (CCV)/Dental Health Service Victoria (DHSV)	Oral Health Policy Data [35, 36] As part of a larger review of health promotion policy CCV conducted a review of oral health municipal promotion plans. Policy and program data was collected by CCV for a project in partnership with DHSV by context analysis of the policies documents which local governments use for promoting the health of their constituents	All local government planning documents across Victoria	Most available data for 2009 Data provided by CCV	Content Analysis
Dental Health Services Victoria (DHSV)	Workforce Dataset [37] Dataset includes the number and type of staff at all community dental clinics in Victoria.	Data is collected at clinic level (all public dental clinics in the State)	DHSV and agency workforce data readily available monthly.	DHSV workforce calculated from the DHSV payroll database Agency workforce data is self-reported by agencies
	Oral Health Promotion Program [38] Smiles 4 Miles is an Oral Health Promotion Program developed by DHSV and is targeted at preschoolers. The data collected is the proportion of kindergartens in a given community implementing the program.	Random sample of kindergartens across Victoria	Data is available from 2009 onwards	Data only represents the number of kindergartens in each region
	Distance to the closest public dental clinic [39] Distance will be calculated using the Victorian census collection districts (CCDs) map. Each CCD will be represented by its centroids which will be imported into a statistical software programme. The distance will be then calculated using the great circle metric.			Distance will be calculated using ‘crow file’ metric which does not take into consideration the ways which patients visit community clinics
Department of Families, Housing, Community Services and Indigenous Affairs (FaHCSIA), Australian Institute of Family Studies (AIFS), Australian Bureau of Statistics (ABS)	Longitudinal Study of Australian Children (LSAC) [25] Longitudinal study following the development of children and families from all Australia. It commenced in 2004 with two cohorts of children. First wave of data collection in 2004 with subsequent main waves every two years The study aimed to investigate the contribution of children’s social, economic and cultural environments to their adjustment and wellbeing	National representative longitudinal sample Cross-sequential design with two cohorts (*N =* 5000 each): 0–1 years and 4–5 years at the commencement of the study Two-stage random clustered design Stratification was used to ensure proportionate number of selected children to the total numbers of children within each state/territory	Accessible data Baseline collection in 2004 and final wave commenced in 2009/2010	Different data collection methods between waves Computer based self-complete questionnaires Sample weights produced to reduce selection bias and participant non-response, validated scales appropriate to children’s age
Victorian Department of Health and Human Services (DHHS)	Victorian Admitted Episodes Dataset (VAED) [40] Episodes of care level morbidity data on all admitted patients from Victorian public and private hospitals including rehabilitation centres, extended care facilities and day procedure centres. The VAED also contains demographic, administrative and length of stay data and data on diagnoses of the patient and medical procedures.	Representative general population level data for all separations of admitted patients from all Victorian public and private hospitals	Ongoing collection Data available after request to the department	Clinical data based on medical diagnosis Extensive validation of data Data are checked for valid values, logical and historical consistency
	Victorian Population Health Survey (VPHS) [27] The VPHS collects information via computer assisted telephone interviews (CATI) at the State, regional and local government levels on health outcomes, determinants and behavioural risk factors of adult Victorians aged 18 years and over	Representative random sample of population aged 18+ residing in Victoria Participation rates vary between years	Annually Accessible data by financial year	Survey data is weighted
	Community Water Fluoridation Program [41] Water fluoridation is the adjustment of the natural amount of fluoride in the water supply to a level recommended for optimal dental health benefits. Some communities in regional and rural Victoria without optimal water receive carefully controlled amounts of fluoride in their drinking water.	Community access to a fluoridated water supply. Coverage is classified geographically by postcode	Accessible data	
	Titanium database [26] The Titanium database is an electronic patient record management system for public dental agencies. It contains administrative, demographic, clinical, oral health and risk factors data.	Eligible population level data only. Eligible is set by government policy and is targeted to populations at highest risk of poor oral health.	Routinely collected Accessible by DHSV staff for all public dental agencies in Victoria	Clinical examination data User data entry error Most common identified areas of inaccuracy include: - Capture of DMF score where dental chart has not been performed or is incomplete - Un-erupted teeth incorrectly recorded as missing inflating the M component of the DMFT score Identification of teeth missing and filled for reasons not due to dental decay
Victorian Department of Education and Training (DET)	Victorian Child Health and Wellbeing Survey (VCHWS) [28] State-wide survey of children 0–13 years of age. The survey is conducted to examine the health of Victorian children and to describe the general health levels and high risk groups. Data is collected on health outcomes, socioeconomic determinants, and behavioural risk factors	Population level cross-sectional survey *N =* 5000, response rate: 86.6 %	Repeated every three years	Parental self-reported data Interviewer training Monitoring and call-back validation Structured questionnaires Existing scales with proven reliability and validity
	School Entrant Health Questionnaire (SEHQ) [29] State-wide survey undertaken when children are in their first year of schooling. The SEHQ contains a range of questions focusing on family demographics, child health and development. The questionnaire is linked to School Nursing program	Population-based level cross-sectional survey of all children entering primary schools	Repeated annually	Parental self-reported data Structured, piloted questionnaire School nurse clinical assessment Existing scales with proven reliability and validity

### Data quality assessment

Table 1 summarises the detailed quality assessment of each of the identified data collections. The majority of the surveys provided incidental information on self-reported outcomes, subject to recall and reporting bias. In contrast, national and Victorian population registries and hospital data are collected routinely, based on medical diagnosis, with large samples, thus the information is generally more reliable. However, their capacity to identify differences in disease profile and service utilisation of population subgroups is restricted, due to limited demographics.

National surveys conducted by the Australian Institute of Health and Welfare (AIHW) and Dental Statistics Research Unit (DSRU) used random sampling design and collected incidental information, with the only exception being the National Dental Telephone Interview Survey (NDTIS) repeated every two and a half years. In contrast, data from state-wide surveys are regularly available. Sampling weights were applied in all surveys. In relation to the Census data, several strategies are employed by the ABS to produce high quality data by minimising respondent errors (choosing suitable content, question and form design), processing errors (repairs, coding errors, and validation), partial response and undercount.

### Data collections and indicators by framework domain

When searching for data sources with items relevant to oral health, and within the domains of the underpinning frameworks, twelve national and eight state-wide data collections were identified.

Oral health status and outcomes indicators were identified in a variety of national data collections including: i) population based surveys: the National Survey of Adult Oral Health (NSAOH) [15], the National Dental Telephone Interview Survey (NDTIS) [16] the Child Dental Health Survey (CDHS) [17], and Child Oral Health Study (COHS) 2002–2004 [17]; ii) administrative systems : the Australian Cancer Database (ACD) [18].

Indicators covering the risk factors and determinants domains were identified in the following national collections: Census of Population and Housing survey [19], Health Care Card data [20], National Health Workforce Data Set (NHWDS) [21],National Hospital Morbidity Database(NHMD) [22],Longitudinal Survey of Dentists’ Practice Activity (LSDPA) [23], National Dental Labour Force Data Collection (NLFDC) [24],and Longitudinal Study of Australian Children (LSAC) [25].

The main state-wide hospital database with oral health status, outcomes, and risk factors items was Titanium [26], a patient record system for Victorian public dental agencies. Data collections with behavioural risk factors were primarily population surveys including theVictorian Population Health Survey (VPHS) [27], the Victorian Child Health and Wellbeing Survey (VCHWS) [28], and the School Entrant Health Questionnaire (SEHQ) [29]. The complete list of identified data collections and indicators is summarised in Table 1.

A total of forty-eight evidence-based indicators of interest were selected across all domains. Majority of determinants and risk factors indicators were within the domains of: i) use of services: dental attendance, reason for attendance, dental general anesthetics (DGA), early detection, preventive care, emergency care and recall period for children; and ii) systems and services: financing care, access to services, waiting time, and organisational practices. Selected oral health conditions and quality of life indicators for measuring outcomes were: caries severity and prevalence, untreated tooth decay, fissure sealants, functional and non-functional dentition, periodontal disease severity, oral cancer mucosal lesions, experience of pain, and psychosocial and functional impacts of oral illness. The list of all selected indicators of interest against the framework domains is summarised in Table 2.

**Table 2:** Summary of identified indicators of interest and available data sources for the Victorian population by framework domain

Domain	Element	Definition of Indicator	Data sources/sets
Determinants & Risk Factors			
Policy	Local government	Proportion of Local Government areas with policies addressing oral health risk factors	CCV & DHSV/Oral Health Policy data
	Setting: Schools and Kindergartens	Proportion of kinder gardens/schools with policies addressing oral health risk factors	DHSV/Smiles for Miles Oral Health Promotion Program
	Health service: Hospitals	Proportion of hospitals with policies addressing oral health risk factors	CCV & DHSV/Oral Health Promotion Policy Data
System and Services	Financing care	Proportion of population eligible for free or low cost public dental services	ABS & DHS/Centrelink
	Access to services	Distance to closest public clinic from census collection district centroid	DHSV/project specific
		Practising dentist per 100,000 population by remoteness category of main practise	AIHW DSRU/National Dental Labour Force Collection AIHW/NHWDS DHSV/Workforce
		Proportion of children in the area who access the public system	DHHS/Titanium
	Waiting period	Waiting times (average period in months) Proportion of population seen within the recommended waiting times	DHHS/Titanium
	Organisational Practices: Recall period for children	Recall period for children (average period in months)	DHHS/Titanium
Environmental	Fluoridated water supply	Proportion of population with access to fluoridated water	DHHS
	Geographical location	Australian Standard Geographic Classification of remoteness	ABS/Census
Socio-cultural	Socio-economic status	Socio Economic Index For Areas (SEIFA)-Index of Disadvantage	ABS/Census
	Education level	Proportion of adult population who did not complete secondary school	ABS/Census VCHWS
	Ethnicity/cultural group	Proportion of adult population who do not speak English at home	ABS/Census
	Household income	Health card holder status	ABS/Census
	Migrant Status	Proportion of population (adults and children) who are migrants	ABS/Census
	Indigenous Status	Proportion of population who are Indigenous	ABS/Census
Use of services	Dental attendance	Proportion of population receiving timely dental care (<12 months)	DHHS/TitaniumDET/VCHWSAIHW DSRU/NSAOH, NDTIS
	Reason for attendance	Proportion of population attending for treatment vs check-up	AIHW DSRU/NSAOH, NDTISDHHS/Titanium
	Dental General Anaesthetics (DGA)	Child DGA rates per 100,000 for removal and/or restoration	DHHS/VAED, AIHW/NHMD
	Early detection/preventative	Proportion of population treated for early disease	DHHS/TitaniumAIHW DSRU/NSAOH
	Care received	Proportion of population who received preventive care	DHHS/TitaniumAIHWDSRU/NSAOH
		Courses of care by provider level: general/emergency/specialist	DHHS/Titanium
	Emergency care	Ratio of emergency to general oral care provided by public oral health care service	DHHS/Titanium
	Recall period for children	Recall period for children (average period in months at when children re-attend the public oral health service)	DHHS/Titanium
Behaviours	Oral hygiene practises	Proportion of children and adolescents who brush their teeth twice a day	DET/VCHWSAIHW DSRU/COHS
	Tap water	Proportion of population who regularly drink tap water	DET/VCHWSDHHS/VPHS
	Diet	Proportion of population who regularly drink sweet drinks	FaHCSIA & ABS/LSACDET/VCHWS
	Alcohol consumption	Proportion of adults who drink alcohol at levels beyond that considered safe in the long term	DHHS/VPHS
	Tobacco	Proportion of population who smoke tobacco	DHHS/VPHS
Outcomes:			
Disease *Children*	Dental caries severity	Mean number of decayed, missing and filled primary (dmft/s) and permanent (DMFT/S) teeth/surfaces with caries experience	AIHW DSRU/CDHSDHHS/Titanium
	Dental caries prevalence	Proportion of children experiencing dental caries (dmft/s > 0 and DMFT/S > 0)	AIHWDSRU/CDHSDHHS/Titanium
	Untreated dental caries	Proportion of children with 1+ untreated dentine decayed teeth	AIHW DSRU/CDHSDHHS/Titanium
	Dental Sealants	Proportion of children with dental sealants	AIHW DSRU/CDHSDHHS/Titanium
	Oral health status	Proportion of children whose parents have concerns about their child’s oral health at school entry	DET/SEHQ
*Adults*	Dental caries severity	Mean number of decayed, missing and filled permanent (DMFT/S) teeth/surfaces with caries experience	AIHW DSRU/NSAOHDHHS/Titanium
	Dental caries prevalence	Proportion of population experiencing dental caries (DMFT/S > 0)	AIHW DSRU/NSAOHDHHS/Titanium
	Untreated dental caries	Proportion of population with 1+ untreated dentine decayed teeth	AIHW DSRU/NSAOHDHHS/Titanium
	Non-Functional dentition	Proportion of edentulous adults	AIHW DSRU/NSAOHDHHS/Titanium
	Functional dentition	Proportion of population with 21+ natural teeth	AIHW DSRU/NSAOH/NDTISDHHS/Titanium
	Periodontal Disease Severity	Proportion of dentate adults with periodontal diseases -severity and extent-	AIHW DSRU/NSAOHDHHS/Titanium
	Oral Cancer mucosal lesions	Annual incidence rates of oral cancer (cancer of the lip, oral cavity, pharynx) per 100,000 population	CCV/VCR, AIHW/ACD
Quality of Life	Experience of pain	Proportion of population who experience tooth ache regularly	AIHW DSRU/NDTIS, NSAOH
	Psychosocial and functional impacts of oral illness	Proportion of adult population often or very often felt uncomfortable with the appearance of their teeth or dentures	AIHW DSRU/NDTIS, NSAOH

Socio Economic Index For Areas (SEIFA): Index developed by ABS that ranks areas in Australia according to relative socioeconomic advantage/disadvantage

A selection of twenty indicators to be included in the model is shown in Table 3, along with the associated data sources. All except for two (caries experience and functional dentition) were determinants and risk factors.

**Table 3:** Summary of indicators to be included in the risk model and associated data sources

Domain	Indicator	Data source/Data Set
Determinants & Risk Factors		
Policy	Local government municipal health plan analysis Proportion of children’s services implementing oral health promotion programs	CCV & DHSV
Systems and Services	Distance to closest public clinic	DHSV/Project specific
	Average time on waiting list	DHHS/Titanium
	Average period of recall time	DHHS/Titanium
Socio-cultural	SEIFA Proportion who are health care card holders	ABS/Census DHS/Centrelink
	Proportion who did not complete secondary school	ABS/Census
	Proportion non-English speaking at home	ABS/Census
	Proportion of migrants Proportion of population who are Indigenous	ABS/Census ABS/Census
Environmental	Proportion without access to fluoridated water	DHHS
	Remoteness	ABS/Census
Behaviours	Proportion of adult (18+ years old) and child (0–13 years old) population who brush their teeth twice a day	DHHS/VPHS DET/VCHWS
	Proportion of adult (18+ years old) and child (0–13 years old) population who regularly drink tap water	DHHS/VPHS DET/VCHWS
	Proportion of child population (0–13 years old) who regularly drink sweet drinks	DET/VCHWS
	Proportion of people who are at long term risk from drinking alcohol (18+ years old) Proportion of population who smoke (15+ years old)	DHHS/VPHS
Use of services	General Anaesthesia (GA) rates Number of courses of care by provider level in each category (general/emergency/specialist)	DHHS/VAED DHHS/Titanium
Outcomes:		
Disease	Dental caries severity	DHHS/Titanium
Quality of Life	Functional dentition	DHHS/Titanium

*AIHW* Australian Institute of Health and Welfare

*DHSV* Dental Health Services Victoria

*SEIFA* Socio-Economic Index for Areas (index of disadvantage)

*VPHS* Victorian Population Health Survey

*VCHWS* Victorian Child health and Wellbeing Survey

*VAED* Victorian Admitted Episodes Dataset

The selection was based on: alignment with the theoretical model, data/item availability and currency, suitability (similarity) of sample population, potential for use in a mixed data-source statistical model. The number of variables from each dataset that have been incorporated into the model varies. Available data is collected at the levels of Local Government Area (LGA), postcode, community dental agency and census collection district (CCD) levels, and where necessary will be disaggregated or aggregated and used at suburb/postcode level to minimise averaging errors. Most recent available datasets for each data collection will be sourced and used.

## Discussion

Identification of communities at high risk of developing dental disease across multiple dimensions enables the appropriate allocation of public dental services and resources, as a first step to reducing oral health inequalities across the population. In the current study, we have identified a guiding framework and multiple data sources to develop a risk assessment model to enable re-orientation of public oral health care services. We have also catalogued available oral health-related data sources to provide a resource for public oral health researchers, policy makers and service providers.

An appropriately directed public oral health service must be able to regularly monitor the costs and clinical effectiveness of the interventions provided, as well as population disease and health inequalities. This requires a shift in the focus and reporting systems from an emphasis on outputs to outcomes. It also requires quality data collections and robust indicator sets. Our research identified numerous sources with items relevant to oral status and outcomes indicators, diseases and related risk factors. These sources were of two main categories: statistical sources, including surveys and censuses, and administrative sources, such as population registries and hospital databases.

The majority of the survey data collected provided incidental information on self-reported outcomes, and were subject to recall and reporting bias. In contrast, population registries and hospital data are based on medical diagnosis thus the information is generally more reliable; however, their capacity to identify differences in disease profile or service utilisation by population subgroups is restricted, due to only limited demographic data being collected. Further, these data collections only include members of the population who utilise such services, and generally do not include individuals who use alternative, privately run services. However, the data sources identified were generally stand-alone and not articulated in with other data collections or monitoring systems. Exploring options for integration of datasets in real time is important to enable more integrated health services to be delivered. Further, improving the data system, so that data linkage at an individual level can occur, will enhance future use of existing data assets, providing a quantum leap in our ability to explore causal pathways and long-term impacts through prospective, longitudinal data sets which are also large and representative. This need, and the potential of this powerful approach for improving oral health, has been recognised by others [30].

Despite the limitations with the data collections, it is clear that there is a wealth of existing survey and routinely collected data that can be better utilised to inform decisions in relation to oral health policy, programs and service delivery. Importantly, we have identified that the available data is not solely located within the health sector which highlights the need for collaborations and partnerships that are cross-sectoral. In this research we have also moved beyond analysing individual items or indicators in data sets, and have identified a robust framework that provides a guiding structure for assembling the available data in a coherent and useful way. This enables us to paint a picture of the risk factors for poor oral health that are operating in the population at a given point in time. Importantly, it is also apparent from this study that no single data collection contains all the necessary information for an integrated oral health-chronic disease surveillance system, as recommended by WHO [9]. Many of the difficulties of using data from multiple and varied sources have previously been identified [31, 32], however the focus has been largely on the appropriate statistical methods to use to provide robust results. There has been little discussion of the practicalities of sourcing, assessing, cleaning, and harmonising data collected at multiple levels such as the individual, family, community and area, although the importance of this for public health has been identified [33].

Data collection is a resource-intensive exercise, and in the absence of a routine oral health monitoring or surveillance system in Australia, a community risk assessment model presents the opportunity to explore patterns and clusters of multiple risk factors in the population based on geographic area. The Model will also enable the examination of multi-dimensional impacts of past and future strategies, interventions and policies on populations, services and systems. This information can then inform health service planning and the approaches to addressing common risk factors and preventing chronic diseases. In the context of limited resources for research and evaluation, having a mechanism to examine population impacts of innovative public health strategies is critical.

### Limitations

This study is limited to the information available at the time of searching. This relates to searching for both frameworks and available datasets. Not all of the data required in the framework could be sourced. It is possible that additional sources are now available. The ability to use the identified data sources in the statistical modelling has not been tested in this phase of the study, which is a further limitation, although this will be the focus of the next stage of the project. We were also unable to find directly comparable studies with which to compare our findings.

### Next steps

The project will continue to progress through the remaining stages shown in Fig. 1. This involves harmonising and combining data sources that were initially collected for different purposes, in different geographical areas, from different subpopulations and/or at different times. Statistical models that have utilised mixed data sources will be scrutinised to inform the best methods to use in the community-level risk assessment model for oral health. Initially the data will be used to investigate the statistical relationship between risk factors and oral health outcomes, to confirm the predictive value of the selected indicators. After exploring and describing the relationships between indicators and outcomes the statistical models of community-level risk will be developed, appropriately including a selection of the indicators of risk. The model deliberately contains both risk and protective factors, as these factors occur concurrently at the community level. This next stage of the project will determine the relative importance of the different influences and provide important, policy-relevant findings. The oral health risk model will be developed for all geographic areas in Victoria and will be used by DHSV to guide resource allocation for public dental services. Routinely collected clinical data will also be monitored to evaluate the effect of utilising a population health planning model for public oral health services on disparities in oral disease across the population.

## Conclusions

The community-level risk assessment model is being developed as a response to a need to transform the approach to reducing disparities in oral health, and re-orient the allocation of public oral health services in contemporary Australia. Given our growing knowledge of the multi-dimensional influences of oral health, and our ability to prevent or arrest the disease with appropriate strategies, there is a clear need to move from a traditional approach to a more meaningful, responsive and effective model for providing public oral health services.

## Abbreviations

ABS: Australian bureau of statistics
ACD: Australian cancer database
AIFS: Australian institute of family studies
AIHW: Australian institute of health and welfare
CCV: cancer council victoria
CDHS: child dental health survey
COHS: child oral health study
DET: department of education and training
DHHS: department of health and human services
DHS: Australian government department of human services
DHSV: dental health services victoria
DSRU: dental statistics and research unit, aihw
FaHCSIA: department of families, housing, community services and indigenous affairs
LSDPA: longitudinal survey of dentists’ practice activity
LSAC: longitudinal study of australian children
NRASL: national registration and accreditation scheme
NDTIS: national dental telephone interview survey
NLFDC: national dental labour force data collections
NHWDS: national health workforce data set
NHMD: national hospital morbidity database
NHWDS: national health workforce data set
NSAOH: national survey of adult oral health
SEIFA: socio-economic index for areas (index of disadvantage)
SEHQ: school entrant health questionnaire
VAED: victorian admitted episodes dataset
VCR: victorian cancer registry
VCHWS: victorian child health and wellbeing survey
VPHS: victorian population health survey
